# Biological Adaptations of Tumor Cells to Radiation Therapy

**DOI:** 10.3389/fonc.2021.718636

**Published:** 2021-11-24

**Authors:** Angeles Carlos-Reyes, Marcos A. Muñiz-Lino, Susana Romero-Garcia, César López-Camarillo, Olga N. Hernández-de la Cruz

**Affiliations:** ^1^ Department of Chronic-Degenerative Diseases, National Institute of Respiratory Diseases “Ismael Cosío Villegas”, Mexico City, Mexico; ^2^ Laboratorio de Patología y Medicina Bucal, Universidad Autónoma Metropolitana Unidad Xochimilco, Mexico City, Mexico; ^3^ Posgrado en Ciencias Genómicas, Universidad Autónoma de la Ciudad de México, Mexico, Mexico City

**Keywords:** radioresistance, radiotherapy, cancer, DNA-damage response, DNA repair pathways

## Abstract

Radiation therapy has been used worldwide for many decades as a therapeutic regimen for the treatment of different types of cancer. Just over 50% of cancer patients are treated with radiotherapy alone or with other types of antitumor therapy. Radiation can induce different types of cell damage: directly, it can induce DNA single- and double-strand breaks; indirectly, it can induce the formation of free radicals, which can interact with different components of cells, including the genome, promoting structural alterations. During treatment, radiosensitive tumor cells decrease their rate of cell proliferation through cell cycle arrest stimulated by DNA damage. Then, DNA repair mechanisms are turned on to alleviate the damage, but cell death mechanisms are activated if damage persists and cannot be repaired. Interestingly, some cells can evade apoptosis because genome damage triggers the cellular overactivation of some DNA repair pathways. Additionally, some surviving cells exposed to radiation may have alterations in the expression of tumor suppressor genes and oncogenes, enhancing different hallmarks of cancer, such as migration, invasion, and metastasis. The activation of these genetic pathways and other epigenetic and structural cellular changes in the irradiated cells and extracellular factors, such as the tumor microenvironment, is crucial in developing tumor radioresistance. The tumor microenvironment is largely responsible for the poor efficacy of antitumor therapy, tumor relapse, and poor prognosis observed in some patients. In this review, we describe strategies that tumor cells use to respond to radiation stress, adapt, and proliferate after radiotherapy, promoting the appearance of tumor radioresistance. Also, we discuss the clinical impact of radioresistance in patient outcomes. Knowledge of such cellular strategies could help the development of new clinical interventions, increasing the radiosensitization of tumor cells, improving the effectiveness of these therapies, and increasing the survival of patients.

## Introduction

Radiotherapy (RT) is an effective treatment against different types of solid tumors detected in early stages, while it is also used as a palliative treatment in metastatic stages. Over 50% of cancer patients are treated with RT and, depending on the type of cancer and the location and size of the tumor, the application can be external or internal (1). The main objective of the RT is to kill tumor cells through DNA damage. However, the damage is detected by tumor cells through a DNA damage response (DDR) mechanism that promotes the activation of cell cycle checkpoints and induces the arrest, or delay, of the cell cycle, inducing the activation of the different DNA repair mechanisms (2). The DDR promotes several cell death pathways, including apoptosis, mitotic catastrophe, necrosis, and necroptosis, activated by death receptors dependent on kinases (RIPK1, RIPK3) (3, 4). The main radiation-activated DNA damage repair pathways are base excision repair (BER), non-homologous end joining (NHEJ), and homologous recombination (HR) (2). However, an increased tumor volume, low oxygen tension, and dysregulation of key genes can lead to tolerance and clonal selection of tumor cells to radiation, thus reducing sensitivity to radiotherapy, leading to tumor recurrence and therapy resistance (2, 5, 6). In addition, the radiation stimulates biological changes like chromatin remodeling, global changes in gene expression, metabolic reprogramming, epithelial-mesenchymal transition (EMT), and disturbances of circadian rhythms, among others (7–14). All changes induced by radiation promote an adaptation biological of tumor cells to the tumor microenvironment, which contributes to aggressiveness and radioresistance of tumors, exacerbating the cancer hallmarks, such as proliferation, migration, invasion, and metastasis (11, 15).

In this review, we describe strategies that tumor cells use to respond to radiation stress, to adapt, and proliferate, promoting the appearance of tumor radioresistance, and highlight strategies that target genes to enhance radiosensitivity in various cancer types.

## Radiation Therapy in Clinical Practice

In clinical practice, radiotherapy (RT) treatment uses two ionizing radiation types: electromagnetic (like X-rays) and Gamma-rays. Radiotherapy aims to kill cancer cells during the treatment. The affected tissues absorb this energy, and its amount applicated is by the unit weight of the organ or tissue and is expressed in units of gray (Gy) (3, 16). Radiation therapy can be delivered externally (teletherapy) or internally (brachytherapy), or both in combination; its use depends on factors such as type of cancer, size of the tumor, tumor location in the body, and regional extent, as well as anatomic area implicated in the geometric accuracy to apply the calculated radiation dose. The efficacy of radiotherapy is established by the therapeutic index of radiation that will be used; this is established by the relationship between the tolerance of the normal tissue surrounding the tumor (NTT) and the lethal dose against the tumor (TLD), whose objective is to erase the tumor and prevent its regression in the affected area (17–20). **Table 1** summarizes the different types of RT currently used in clinical practice and their advantages and disadvantages.

**Table 1:** Types of radiotherapy used in clinical practice for the treatment of different types of cancer.

Teletherapy (applied externally)						
Protocol type	Characteristics	Cancer treated	Example of protocol	Advantage	Disadvantages	References
Three-dimensional conformal radiotherapy (3D-CRT)	Radiation administered geometrically from the volume to be treated	Prostate, spine, esophagus, lung, bladder, pancreas, head and neck cancer	Adjuvant (additional to chemotherapy) for locally advanced non-small-cell lung cancer; 55 to 65 Gy administered in three sessions over approximately 4 weeks	Uses three-dimensional images for the geographic location of the tumor Radiation beam is tailored to target tumor Limits radiation dose to adjacent tissues	Requires very precise dosing and planning to minimize exposure of surrounding normal tissues to radiation dose Requires specialized equipment Long treatment	(17, 21–23)
Intensity-modulated radiation therapy (IMRT)	Controls the shape (similar to 3D-CRT) and also the intensity of each beam emitted Reduces the exposure of healthy tissue to radiation	Prostate, spine, lung, breast, kidney, pancreas, liver, tongue, and larynx cancer	In prostate cancer (PCa), 62 Gy in 20 fractions, over 4 weeks	Dose limitations of the target tumor and adjacent tissues Vary dose intensities in the treatment field	Requires very precise doses Long treatment Requires specialized equipment	(17, 21, 24, 25)
Stereotactic Body Radiation Therapy (SBRT) or Stereotactic Ablative Radiation Therapy (SABR)	Uses multiple beams of radiation, from many different directions, that converge into a very small volume Allows high doses of radiation to be delivered with little damage to surrounding healthy tissue	Used in the treatment of small tumors in the head and brain, also in lung, spine, and liver cancer	In PCa, 25 Gy in five fractions over the course of 1–2 weeks	Precise geographic location of the tumor Use high doses The treatment can be completed in a few fractions (1 to 5) and in a short time (1 to 5 days) Improves response to treatment Can be used in combination with chemotherapy A treatment for inoperable tumors	Difficult to manage Requires a lot of pressure Requires specialized equipment	(21, 24, 25)
Volumetric modulated arc therapy (VMAT)	Supplies the radiation beams by means of a 360° arc integrated into a linear accelerator Treatment cycles are very fast (less than 2 min) Provides very high doses of radiation with precision and speed.	In head and neck tumors, PCa, or central nervous system tumors.	Twenty Gy in a single dose for the treatment of brain metastasis	Radiation adapts to the shape of the tumor to minimize exposure to healthy structures Rapid treatment administration	Longer doses	(21, 26–28)
**Brachytherapy (Applied internally)**						
**Protocol type**	**Characteristics**	**Cancer treated**	**Example of protocol**	**Advantage**	**Disadvantages**	**References**
Interstitial	Administration within the tumor	Uterus and recurrence of vaginal cuff cancer	In uterus cancer, three or four 6 Gy fractions, one fraction per week	High doses in tumor and low in healthy tissue Allows the treatment of larger tumors	Invasive Formation of necrotic cavities	(29, 30)
Intracavitary	Administration inside a natural (as vagina or larynx) or surgically created cavity	Larynx, uterine, cervical, and endometrial cancer	In cervical cancer, 15 or 20 Gy in three or four fractions.	Uses anatomical pathways to place radioactive sources Can be used without anesthesia You can use low dose, pulsed dose, or high dose	Higher risk of error	(31, 32)
Intraluminal	Application into the lumen of organs	Extrahepatic biliary duct cancer and esophagus cancer	For biliary duct cancer, 30 Gy for definitive dose	High doses of radiation to the tumor and minimize the dose to healthy adjacent organs Allows biliary drainage through the tumor Improve survival	May cause bleeding	(33, 34)
Intravenous	Venous administration of radioactive molecules	Hepatocellular carcinoma	For hepatic cancer, 100 Gy in a single dose	Little invasive Quick and easy administration Therapy targeting specific proteins on the surface of tumor cells	Long treatment May cause side effects	(35)

## Tumor Cells Activate Signaling Pathways Involved in DNA-Damage Response to Survive Ionizing Radiation

Despite the recent technological advances in treatments against cancer, some tumors develop acquired resistance or have intrinsic resistance, which is a problem in the fight against cancer (36). In addition, the tumor heterogeneity can promote innate response favorable to radiation. However, the tumor heterogeneity induces the development of intratumoral resistance to radiation through clonal selection (37, 38).

Ionizing radiation (IR) produces DNA lesions, among them double-strand breaks (DSBs), the most lethal form of DNA damage, and single-strand breaks (SSBs). Ionizing radiation can, directly and indirectly, damage DNA, causing ionization of the atoms or breaking its bonds in the DNA molecules or by the production of highly reactive free radicals, which can interact with the DNA. DNA damage by exogenous agents like radiation is sensed by DNA damage response (DDR), mediated by activation of the DNA repair pathways (12, 18).

**Figure 1:**
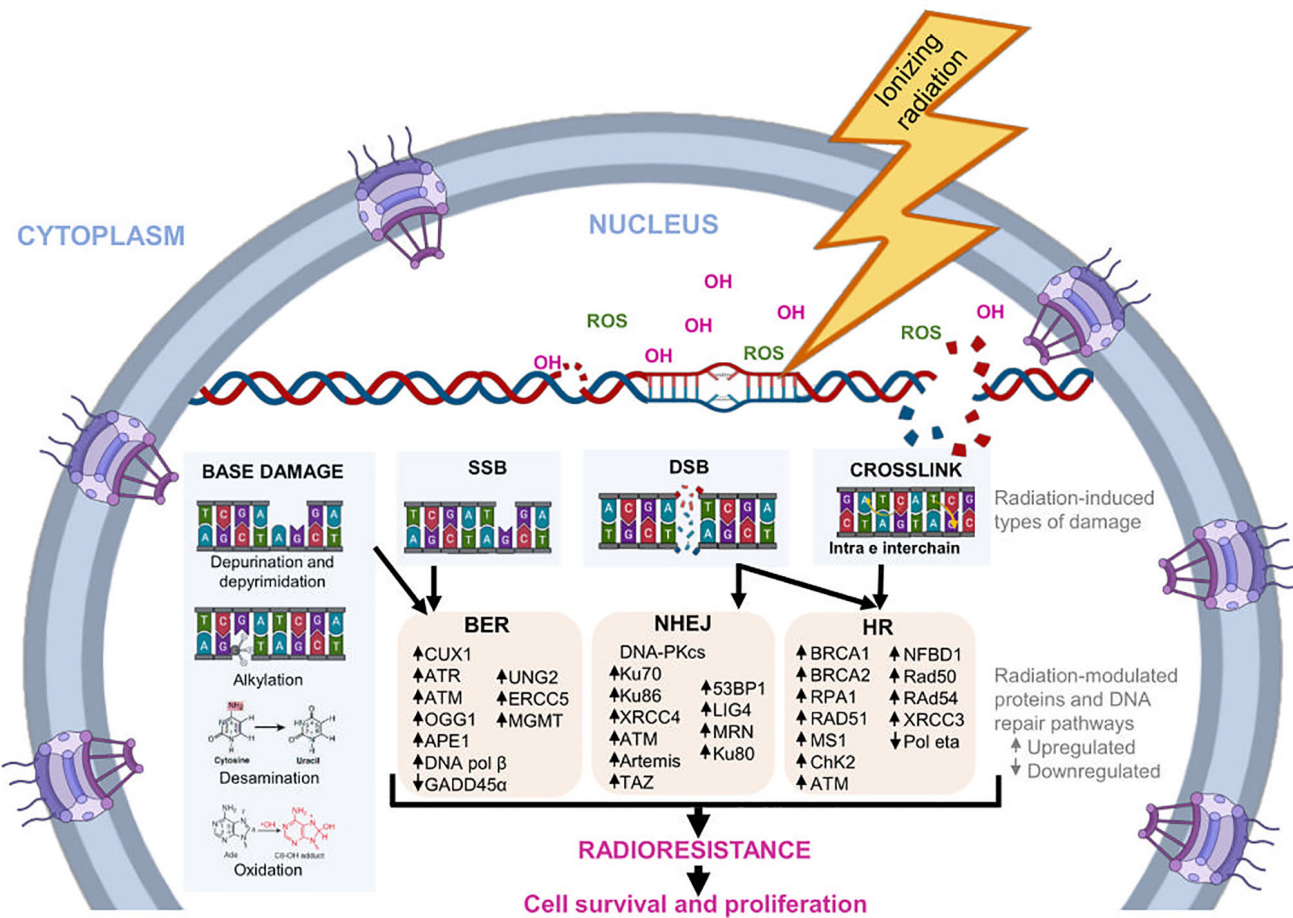
DNA repair pathways induced by radiation. During radiotherapy, IR can alter the chemical structure of DNA directly or indirectly. Indirectly, it promotes the formation of molecules, such as the OH- ion and ROS, which bind to nucleotides and modify them structurally. The main modifications induced by radiation are base damage, crosslink, SSB, and DSB. In response, cells regulate the expression of several genes and proteins involved in different DNA repair pathways, such as BER, NHEJ, and HR. The activation of this pathways helps to reduce radiation-induced DNA damage, favoring the survival and proliferation of tumor cells, as well as cellular radioresistance.

DDR also induces the cell cycle arrest through regulation checkpoint kinases and promotes apoptosis when DNA damage repair mechanisms fail (39, 40). Damage to DNA is repaired by activation of various repair pathways, like base excision repair (BER), non-homologous end joining (NHEJ), and homologous recombination (HR) (41, 42). **Figure 1** shows some proteins involved in DNA repair pathways modulated in response to radiation.

### BER Pathway

This mechanism can repair more than 90% of radiation-generated DNA damage, which includes injuries on nitrogenated bases caused by oxidation, alkylation, deamination, and depurination, as well as SSBs (43). Briefly, this repair route detects and removes damaged bases through specialized DNA glycosylases, which are constantly scanning the damaged DNA. The UNG glycosylase hydrolyzes the N-glycosylic bond between the DNA base and sugar-phosphate backbone to produce a basic site. Then, APE1 endonuclease cleaves the phosphodiester bond to generate an SSB. DNA polymerase β (pol β) acts as an AP lyase, removing the sugar attached to the 5′ phosphate, and DNA polymerase adds nucleotides to the end of SSB. Finally, a DNA ligase seals the nicks (44).

It has been reported that key factors for the BER pathway are overexpressed or activated in radioresistant cells. For example, the CUX1 transcription factor is overexpressed in colorectal cancer (CRC) cell lines that exhibit high levels of ROS and is required for the activation of DDR using multiple transcriptional targets, such as ATR and ATM (45). In addition, CUX1 stimulates OGG1 expression, a DNA glycosylase involved in removing oxidative purine lesions (46). Naidu et al. found that cells with higher endogenous APE1 endonuclease are more radioresistant, and the APE1 ectopic expression in glioma cell lines has a dose-dependent effect, increasing radioresistance (RR) (47). Low expression of GADD45α, an APE1-binding protein, has been observed in radioresistant cancer cells and biopsies from radioresistant cancer patients. Li et al. reported that GADD45α subexpression protects from radiation-induced cell death and DNA damage contributing to the development of RR in cervical cancer (48).

On the other hand, Nickson et al. note that oropharyngeal squamous cell carcinoma (OPSCC) patients that are HPV-16 positive (+) have the most radiotherapy treatment sensitivity and survival, while HPV-16 negative (−) OPSCC patients have a lesser response to the same therapy. In addition, in *in vitro* studies in cell lines, HPV-16-positive cells and HPV-16 negative cells showed a relationship similar to that observed in OPSCC patients (HPV-16+/HPV-16−), related to the low efficacy of DNA repair mechanism in HPV-16 (−). Additionally, OPSCC HPV-16+ radiosensitive cells express high levels of the XRCC1, DNA polymerase β, PNKP, and PARP-1 proteins related to the BER and SSB repair mechanisms. At the same time, treating HPV-16 (−) cells with a PARP inhibitor (olaparib) and radiotherapy induces the most therapy radiosensitivity. The radiotherapy response is most effective in HPV-16 positive OPSCC patients compared to HPV-16 negative OPSCC patients (43). DSB is the most complex and lethal type of DNA damage. When DSBs occur during RT, proteins involved in the NHEJ and HR pathways are turned on to promote the survival of tumor cells against damage.

### The NHEJ Pathway

In mammalian cells, most DSB lesions are repaired by the NHEJ pathway. This is a mechanism triggered in cells in any phase of the cell cycle and allows DSBs to rejoin. The initial step in the NHEJ pathway is to recognize and protect free DNA ends by Ku70/80 heterodimer. After, the Ku70/80 complex recruits additional members of the NHEJ pathway to the damage sites, such as DNA-PKcs, forming the complex know as DNA-PK. DNA-PKcs, activated by autophosphorylation or ATM, phosphorylates different factors required for DNA end-processing, including Artemis endonuclease, Mre11/Rad50/Nbs1 complex, and different polymerases. Finally, DNA ligase 4 (LIG4) is responsible for catalyzing the ligation of the DNA ends (49).

In radiotherapy-resistant prostate cancer cell lines (PC3, DU145, and LNCaP), the DNA damage by radiation promotes DSBs mediated by DNA NHEJ and HR repair mechanisms activation, increasing Ku70, Ku80, BRCA1, BRCA2, and Rad51 expression of proteins, respectively. The resistant cells showed cell cycle arrest in G0/G1 and S phase through an increase in p-p53 (p53 phosphorylated) and p21 by Chk1/2 activation. Besides, the activation of caspase 3 and 7, the decrease of PARP-1 and Bax protein expressions, as well as the expression high of Bcl-2 and Bcl-xl proteins promote the inhibition of apoptosis, as well as autophagy, through the increased expression of Beclin-1 and LC3A/B (50). In another report, Beskow et al. showed an increased expression of genes involved in NHEJ (such as DNA-PKcs, Ku70, and Ku86) in the residual carcinoma from patients with cervical cancer after RT relative to corresponding primary tumors (51). Accordingly, low expression of Ku80 in cervical cancer patients also shows a better response to RT, and therefore a greater overall survival of patients (52). In agreement, low expression of Ku70 or XRCC4 proteins in hypopharyngeal squamous cell carcinoma patients was related to better locoregional control, suggesting a greater sensitivity to chemoradiotherapy (53). TAZ is a transcriptional coactivator upregulated in different types of cancer; its overexpression stimulates the expression of genes involved in NHEJ, such as TP53BP1 (53BP1), PRKDC (DNA-PKCs), and XRCC6 (Ku70), contributing to the radioresistant phenotype. It has been associated with clinicopathological features, poor prognosis, and radioresistance in esophageal cancer cells. Furthermore, TAZ overexpression increases various hallmarks of cancer, such as proliferation, migration, invasion, and decreased apoptosis (54).

*In vitro* studies have shown that radiation modulates the expression of different proteins involved in NHEJ. Bian et al. established a radioresistant breast cancer cell line (MD-PR) through prolonged and repeated exposure to radiation. After radiation, MD-PR presented higher expression of phosphorylated ATM and ATR than parental cells, resulting in higher efficiency in DDR and NHEJ. On the other hand, Artemis is rapidly hyperphosphorylated by ATM in response to radiation and subsequently recruited to the damaged sites together with 53BP1 to coordinate the binding of the DSBs (7). Other radiation-modulated proteins are DNA ligase IV (LIG 4) and TAZ. LIG 4 senses DSBs and facilitates cell survival following treatment with ionizing radiation. Lung cancer cells (LCCs) expressing mutant LIG4 are sensitive to ionizing radiation (55, 56). Additionally, the C-X-C motif chemokine ligand 1 (CXCL1) oncogene secreted by components of the tumor microenvironment is highly expressed in various cancer types, promoting tumor angiogenesis, migration, invasion metastasis, tumor progression, and chemoresistance (57). In esophageal squamous cell carcinoma, the cancer-associated fibroblasts (CAFs) were found to produce high expression of chemokine CXCL1, which promotes radiotherapy resistance *in vitro* and *in vivo* in ESCC through an overregulated expression of DNA damage repair proteins (e.g., p-ATM, Rad50, p-Chk2, Ku80, and DNA-PKcs) and the Mek/Erk signaling pathway activation, as well as an increase of γ-H2AX protein. Besides, CXCL1 inhibits the expression of superoxide dismutase 1 (SOD1) and induces the accumulation of ROS-induced DNA damage repair pathways (27). In glioblastoma (GBM), the high expression of CXCL1 was related to poor prognosis of patients induced radiotherapy resistance through EMT event and using activation of NF-κB signaling (58).

### The HR Pathway

HR is a complex pathway specifically triggered in later-S and G2/M phases of the cell cycle because a homologous sequence is used as a template to restore dsDNA breaks, DNA gaps, and DNA interstrand cross-links. Compared with the NHEJ pathway, HR is a process that provides high-fidelity, requires more proteins to repair, and reduces the probability of genome rearrangements and loss of genetic material. During HR, DSB ends are recognized and resected by nucleases (Mre11-Rad50-NBS1 complex, Exo1, Dna2, Sae2/CtIP) and a helicase (Sgs1/BLM) to form a terminal 3′-OH single-stranded DNA tail. Then, the RPA protein binds to the tail and inhibits the formation of secondary structures in the ssDNA chain. Rad51 recombinase is recruited onto ssDNA through mediator proteins and forms a nucleofilament called the presynaptic filament. The Rad51 nucleofilament must search the homologous sequence located in the intact sister chromatid and invade (synapsis), generating the displacement of the homologous DNA strand to form the so-called D-loop. After D-loop formation, the invading chain is elongated by a polymerase, thus synthesizing the information lost during the DSB, then released. Later, multiple subpathways can be used for the resolution and repair of the DSBs (59–61).

Multiple studies have shown that radioresistant cells have an increase in DNA repair by HR compared to radiosensitive cells (43, 62). In breast cancer, the treatment used for conserving of breasts (BCT) is surgery plus adjuvant radiation therapy. However, some patients experience tumor recurrence around the scar. In the use of intraoperative radiotherapy (IORT) with intensive radiation administered during surgery directly to the tumor bed while sparing normal surrounding tissue, it has been observed that IORT induces biological changes in the tumor microenvironment and the activity of surgical wound fluid (RT-SWF) of breast cancer. These RT-SWF promote a DDR in the MDA-MB-468 cells, inducing overregulation of ERCC2, ERCC8, and RAD51 of the repair mechanisms NER and HR, promoting the arrest cell cycle at the G2M phase and raise its glycolytic metabolism (63). Overexpression of BRCA1/BRCA2/RAD51/RPA1 proteins in the HR was detected in hypopharyngeal carcinoma cell radioresistance, promoting the S phase and G2 phase cell cycle arrest. However, the RPA1 deletion in these cells leads to sensitivity to radiation (64). A similar study in a nasopharyngeal carcinoma (NPC) (CNE2RR) cell line induced the expression of NFBD1, BRCA1, BRCA2, RPA1, and RAD51 proteins widely associated with HR and radioresistance (65). Another report on this cancer found that the interaction of RAD50 (recombinant) with Mre11 and Nbs1 leads to G2/M cell cycle arrest through decreased DSBs, inhibits colony formation, and promotes sensitivity to radiation (66).

High expression of MSI1, CHK2, and Rad51 and higher ATM phosphorylation was reported in radioresistant stem-like cells from patient-derived glioblastoma (GBM). Furthermore, the overexpression of MSI1, a stem-like marker, promoted an increase in survival, invasion, EMT-like phenotype, and maintenance of cancer stem properties after radiation, through hyperactivation of DDR and DNA repair by HR (67). Some DNA lesions may persist despite the efficient activation of the different repair pathways in response to damage. In these cases, the cells can turn on the translesion DNA synthesis (TLS) mechanism, where a low-fidelity polymerase (such as Pol eta) induces a bypass of DNA damage to ensure continued genome duplication and cell survival. Paradoxically, irradiated cells lacking Pol eta showed greater radioresistance and survival through inhibition of the TLS mechanism, increasing the number of DNA templates and stimulating DNA repair by HR (68).

DNA repair pathways can compete or work together and converge at some point because, potentially, all types of damage can be generated during the irradiation. However, many details are still unknown (69). For example, leukemia cells lacking DNA pol β cannot perform the BER pathway efficiently but can activate the NHEJ pathway to repair damage by alkylation (70).

Cells have developed multiple pathways to detect DNA damage and coordinate the response to DNA damage, so the cell fate (survival or death) depends on their ability to activate these pathways quickly and efficiently. After irradiating HT29 colon cancer cells, ATM is activated by phosphorylation, promoting the recruitment of multiple factors involved in DDR, such as MDC1 and 53BP1, into the γ-H2AX repair foci (71). Chk2, a DDR regulator activated by ATM in response to damage, interacts with p53 to modulate the cell cycle (72). DSBs also stimulate the activation of GSK-3β by ATM. Subsequently, it is translocated from the cytoplasm to the nucleus, where it participates in the recruitment of other repair factors to the site of damage. Examples of these factors involved in NHEJ are 53BP1 and MRN and UNG2 involved in BER (59). WNT proteins are overexpressed and activated by radiation and promote RR in several human cancers, such as CRC and intestinal stem cells through the Wnt/β-catenin signaling pathway. After irradiation, β-catenin is stabilized by Wnt; it is translocated to the nucleus, enhancing the expression of different gene targets, such as LIG4 (73). The Wnt canonical pathway has also been associated with survival and aggressiveness of tumor cells after radiation because it promotes the maintenance of CSCs, EMT, and apoptosis evasion, contributing to RR and relapse of cancer (74).

The hippo pathway has an important role in regulating cell proliferation, organ growth, and cell regeneration. It has been reported that this occurs *via* a pivotal role in cell growth, invasion, metastasis, and its components could be therapeutic target potential in cancer (75, 76). In a glioma U251 cell line, irradiation induced cell apoptosis through high expression of c-caspase 3, caspase 3, and Bax. Irradiation also promoted a low expression of YAP and the inactivation of Hippo/YAP signaling through the ubiquitination mediated by RCHY1 ubiquitin ligase, as well as the high expression of Mst1, LATS1, MOB1, and SAVI (77). Whereas the medulloblastoma cells were irradiated, a YAP high expression was detected, which induced the cell proliferation through high-rise Cyclin D2 (CCND2), and phosphorylated H3 promoted the tumor aggressiveness and tumor recurrence. Besides, YAP promotes IGF2 expression, which promotes the activation of PI3K/Akt pathway signaling. Akt activity automatically induces ATM and Chk2 dephosphorylation, immediately the lock of the DDR mechanism, thereby favoring radioresistance (78).

The Brahma-related gene product 1 (BRG1) enzyme catalyzes the SWI/SNF chromatin remodeling complex activity. BRG1 participates in proliferation, migration, and cellular and cell cycle progression in cancer (79). BRDs are conserved molecules that bind the acetylated lysine residues of the histone tails, leading to the regulation of gene expression, participate as readers of chromatin state, and repair DNA damage by activating DDR mechanisms. In cancer, BRDs are dysregulated, promoting the cell cycle and metastasis (80, 81). In colon cancer, BRG1-BRD dimerization was detected to have a greater chromatin binding strength, leading to radiosensitivity through γ−H2AX foci formation block and DSB repair. Also, this interaction inhibits the accumulation of 53BP1 towards the DSB sites and no alteration of ATM, CHK2, and p53 activations (71). On the other hand, in radiotherapy-resistant cervical cancer cell line (HeLa), the expression of DNA Damage-inducible protein 45α (GADD45α) was detected, promoting the increase cytoplasmic APE1 levels in these cells through overregulation of nitric oxide (NO), and inducing the nuclear export of APE1 to the cytoplasm, promoting cell proliferation and inhibiting apoptosis (48).

DSBs are the most lethal type of DNA strand damage and constitute the most complex type of damage. Consequently, it has been extensively studied. When DSBs occur, two evolutionarily highly conserved repair pathways are turned on: NHEJ and HR. In the same way, factors involved in both repair pathways are key to promoting tumor cells’ survival against radiation damage.

## Cell Cycle Adaptations in Response to Radiation

During the cell cycle, the cell duplicates its genome, grows, and divides; these events are regulated through cyclin‐dependent kinase (CDK) in the checkpoints in the phase difference. Loss of cell cycle control is one of the hallmarks of cancer (82). The biological alterations in the cell cycle by radiation show changes in the phases of the cell cycle; for instance, in cervical cancer cell line HeLa irradiated with Gy (Gray) x-ray in different doses was observed an important G2/M retardation of these cells, decreased CDK1 protein expression, and increased CHK1 expression. Furthermore, the radiation promotes DNA damage by DSBs and a high γ-H2AX expression and production of ROS after radiation (83). Other research, in an oral cancer cell line SCC4 treated with RAD001 (an inhibitor of mTOR) plus radiation, reduced mTOR-S6 and 4EBP1 activation was detected, as well as the arrest in the G2/M cell cycle phase. This phenomenon was induced through CHK1 activation due to phosphorylation in Ser345 position and inhibition of CDC2-cyclin B1 complex and high levels of histone H2AX phosphorylation, thus inhibiting the proliferation of these cells (84). On the other hand, Chang and coworkers showed that PI3K/Akt/mTOR signaling pathway inhibitors (BEZ235 or PI103), in combination with radiotherapy in resistant prostate cancer cell lines (PC-3RR, DU145RR and LNCaPRR), promote distribution of cell cycle toward (G2/M) phase and decrease of G0/G1 and S phases through reduced protein phosphorylation of p-CDK1, p-Chk1, p-Chk2, and p-Rb. Moreover, apoptosis was induced by activating caspase-3 and caspase-7, with the split-off PARP-1, high γH2AX expression, and a decrease of repair proteins Ku70 and Ku80 BRCA-1, BRCA-2, and Rad-51 of NHEJ and HR, respectively, increasing to radiosensitivity in this cancer (50). Multiple studies have reported that tyrosine phosphatase (SHP1) is a negative regulator of cancer cell proliferation, EMT, migration, invasion, and cell cycle (85). In lung cancer, resistant cell lines (A549S1 and S549S2) show high levels of expression of SHP1, CDK4, and CylinD1 and low p16 expression. SHP1 promotes resistance to radiotherapy through regulating G0/G1 phase arrest of the cell cycle (86).

In another study, comparing two methods of radiation, one with carbon ions and the other X-irradiation in prostate cancer and colon cancer (PC3 and Caco-2 cell lines), it was observed that the carbon ions induce a higher γH2AX foci formation in colon cancer than in prostate cancer. X-radiation promotes lesser γH2AX foci formation, which is dose dependent, in both types of cancer. Furthermore, low doses of carbon ions trigger the G2/M arrest phase continuously, whereas high doses of radiation-X can keep the G2/M arrest phase in these cell lines and promote radiosensitivity (87). Radiotherapy promotes accumulation in the G2/M phase of the cell cycle in the different cancer types.

## Chromatin Remodeling as a Mechanism of Radiation Adaptation

The genome of eukaryotes is located in a highly compacted core in chromatin form; this is a dynamic structure that maintains genomic stability and regulates gene expression and DNA repair. Chromatin remodeling is done through covalent modification of histones and the catalytic activity of remodeling proteins (88). For more than two decades, it has been reported that structural changes in the chromatin organization can contribute to the RR of tumor cells (89). The chromatin supercoiled (heterochromatin) configuration is more susceptible to developing radiotherapy resistance than the relaxed chromatin (euchromatin) of tumor cells (90). For instance, in colon cancer, heterochromatin formation and histones methylation were observed in the irradiated subpopulation of cancer stem cells; both could promote radioresistance in this cancer (91).

Another study in a lung cancer cell line and head and neck squamous carcinoma cell line reported that more condensation of heterochromatin of irradiated cells is observed in 3D cultures than with 2D cultures, through decreased histone H3 acetylation and HP1a expression and fewer DSBs, promoting resistance toward radiotherapy (92). It has been described that genome compaction is a protective mechanism deployed by irradiated cells to protect the integrity of DNA against ionizing and other damaging agents. Takata et al. demonstrated that after γ-irradiation, the frequency of occurrence of DSBs is 5–50 times less in compact chromatin than in decondensed chromatin. However, they observed that this effect is a consequence of a lower chromatin opening rather than an increase in the concentration of associated proteins (14, 93).

Interestingly, the protective effect extends to other irradiation sources, such as carbon ion (C-ion), and chemical agents, such as cisplatin, both used in cancer therapies. Consistent with this, Sato et al. observed that cells subjected to different doses of X-rays can develop RR not only to X-rays but also to C-ion. It has also been reported that resistant C-ion cells may be sensitive to X-rays. These data suggest that resistance mechanisms to different sources may overlap. In the same report, they showed for the first time that the degree of RR correlates directly with the number of heterochromatic domains present in cells, so this characteristic could be used as an indicator of RR (14, 93).

Mund et al. (94) reported that after γ-irradiation of human bone osteoblastoma cancer cells, SPOC1 protein is recruited to DSBs-repair foci in an ATM-dependent manner. At repair sites, SPOC1 interacts with chromatin and chromatin remodeling factors, stimulating heterochromatinization and DDR (94).

Wang et al. also reported that EGFR is another protein involved in chromatin compaction after radiation in non-small-cell lung cancer (NSCLC) cells, and its inhibition can induce cellular senescence, increase the number of DSB, and radiosensitization, so it has been proposed as a therapeutic target for this cancer (95, 96).

The formation of highly condensed and ordered chromatin can reduce the exposure of DNA to OH and ROS radicals and decrease the direct ionization of DNA, thus increasing cell survival. On the other hand, heterochromatinization can promote the DNA repair activity of tumor cells through a greater restriction in molecular diffusion and thus promotes the detection of lesions. The latter is of great importance during HR repair since the colocalization and stability of the sister chromatids and the mechanical components are favored for rapid and accurate rejoining. The compaction of chromatin in response to radiation, and other stressors, has been reported in several species, so it appears to be a highly conserved adaptation mechanism (97).

However, highly compact chromatin constitutes a barrier limiting the access of proteins that participate in DDR to DNA damage. Therefore, regions to repair must be locally reconfigured towards more relaxed chromatin to promote efficient repair and after repackaged again into nucleosomes (98). Chromatin remodeling proteins facilitate the recruitment of essential factors required during DNA repair. Brahma-related gene-1 (BRG1), the central catalytic subunit of many chromatin-modifying enzymatic complexes such as SWI/SFN, has been implicated in the ATP-dependent local alteration of chromatin structure after radiation. After DSBs formation, the ATM protein is activated and phosphorylates H2A histone family member X (H2AX) located at the damage sites, resulting in the formation of γ-H2AX-containing nucleosomes. Subsequently, BRG1 is recruited to damage sites through its interaction with acetylated histones H3 of γ-H2AX nucleosomes, where it promotes the disruption of histone-DNA contacts, thus increasing the local accessibility of DNA to repair proteins, stimulating DDR and apoptosis evasion (71, 99). On the other hand, Andrade et al. reported that by protein-RNA interactions in breast cancer cells, HuR stabilizes the ARID1A mRNA, a subunit of the SWI/SNF chromatin remodeling complex, reducing radiation-induced DNA fragmentation, possibly through NHEJ pathway stimulation, thus reducing DSBs accumulation and conferring RR (100).

## Changes in the Plasma Membrane That Favor Radioresistance

The plasma membrane is a semipermeable lipid bilayer associated with different proteins and carbohydrates; their composition and organization largely determine its role within different biological processes. The plasma membrane helps maintain cell homeostasis by serving as a barrier between the intracellular and extracellular environment, regulating the transport of molecules, and is involved in cell communication and cell signaling in cell movements. After radiation, tumor cells can alter the expression of genes that promote changes in the composition of lipids and membrane proteins, thus promoting their reorganization and increasing the RR phenotype (101).

Astrocytoma cells can rearrange their plasma membrane and form thin and ultralong (up to hundreds of micrometers) protrusions, also called tumor microtubes (TMs), in response to radiation. The formation of these TMs may support brain invasion, proliferation, and multicellular communication over long distances; importantly, TMs-interconnected tumor cells were more resistant to RT. On the other hand, an increase of intracellular calcium has been reported to promote the sensitization of tumor cells to radiation (101). Osswald et al. have reported that intracellular calcium levels increase in cells that TMs do not connect after radiation. However, cells interconnected by TMs present more homogeneous calcium levels, similar to those of non-irradiated cells. The formation of TMs favors the cellular interconnections and the maintenance of calcium homeostasis since they could serve as bridges for the distribution and homogenization of calcium between cells and protect cells from cell death. Few proteins involved in the formation and function of TMs have been identified; one of them is the growth-associated protein 43 (GAP-43), a protein associated with neuronal growth and plasticity. After radiation, GAP-43 is overexpressed, stimulating TMs formation, increasing cellular interconnectivity, and forming a communication network within the tumor (102). By gene-expression microarray analysis, Jung et al. identified the Tweety-homolog 1 (Ttyh1) protein as a new TM-relevant factor. Ttyh1 is a plasma membrane protein associated with neuronal development that colocalizes with integrin α5 and is highly expressed in invasive cells with one or two TMs, compared to less invasive cells with more than two TMs. However, although Ttyh1 expression is important for TMs formation, Ttyh1-deficient cells with more than two TMs showed higher TMs interconnectivity, leading to increased RR of tumor cells (103).

Both reports agree that radioresistant tumor cells presented more interconnecting TMs. In breast cancer, Chignola et al. reported that the formation of intercellular cytoplasmic bridges and the presence of multinucleated giant cells increase in response to radiation and significantly stimulate tumor RR. An increase in cytoplasmic bridges formation, and greater communication between cells within a tumor population, is stimulated by the action of Syncytin-1 homologous protein (SyHP). Syncytin-1 is a viral protein involved in fusogenic events between viral and cell membranes. After radiation, a portion of the cell population begins to die, exposing the SyHP protein on its surface. SyHP exposure on dead cells serves as a stimulus for the formation of cytoplasmic bridges and the induction of fusion events between the surviving cells, resulting in syncytia formation and increase of the tumor population survival (104). In CRC cell lines, the radiation triggers plasma membrane alterations, such as loss of polarity, spindle-cell shape, intercellular separation, and the emergence of pseudopodia; these changes increase invasion, migration, and survival of the radiated cells (105).

In the plasma membrane, ASMase hydrolyzes sphingomyelin generating ceramide; this process is carried out especially in lipid rafts, sphingomyelin-rich membrane microdomains involved in cell signaling. Ceramide-rich lipid rafts rearrange and fuse, forming large lipid platforms (106, 107). Ketteler et al. showed that stress by radiation stimulates changes in the lipid composition of plasma membranes, promoting their reorganization, altering downstream cell signaling, and affecting the RR of PCa cells. After radiation, epithelial cells (EC) stimulate the activation and translocation (from the lysosome to the plasma membrane) of the ASMase enzyme and decrease the expression of caveolin-1 (CAV1), increasing apoptosis. However, CAV1 overexpression has been reported in malignant EC of different types of solid tumors; tumor cells could increase CAV1 expression as a mechanism for evasion of apoptosis and RR (108).

## Endoplasmic Reticulum Adaptations to Radiation

The endoplasmic reticulum (ER) is an endomembrane system that participates in multiple cellular functions, mainly related to synthesis, folding, modification, and transport of proteins (109). Radiation and chemotherapeutic drugs can perturb cellular homeostasis and generate stress in the ER; numerous evidences indicate that said stress (ERS) plays an important role in activating resistance mechanisms to radiation and drugs (110). The accumulation of unfolded or misfolded proteins in ER lumen after radiation activates a cytoprotective unfolded protein response (UPR) that maintains ER homeostasis. However, the UPR pathway can induce cell death if stress is severe and persistent (13).

RR in oropharyngeal carcinoma cells (OPCCs) is regulated by protein kinase R-like endoplasmic reticulum kinase (PERK), one of the main sensors and transducers of the ERS pathway. After radiation, PERK is autophosphorylated and phosphorylates to the eukaryotic initiation factor-2 (eIF2α) factor, which subsequently inhibits the global synthesis of proteins, reducing translocation and accumulation of misfolded proteins in the ER lumen. At the same time, phosphorylated eIF2α activates NF-kB, which is translocated to the nucleus and promotes the transactivation of its target genes. This process inhibits G2/M cell cycle arrest and apoptosis, as well as stimulates DNA DSB repair (110). Additionally, NF-κB confers RR in lymphoma cells by, at least in part, inducing the aberrant expression of HIF-1 (111). IRE1 is another principal sensor of ERS pathway, and its overexpression in HPV-negative OPCC patients treated with RT has been correlated with poor outcomes. IRE1 promotes IL-6 activation, enhancing X-ray-induced DNA DSB and cell apoptosis (112). Another mechanism that activates ERS signaling is the activation of EGFR conferring RR in OSCC. The EGFR inhibition improves therapy in non-response OPCC patients by inhibiting PERK-eIF2α-GRP94 and IRE1α-GRP78 (113).

The ERS pathway also stimulates chaperones’ expression to assist protein folding; the chaperone glucose-regulated protein 78 (GRP78) has been reported to increase its expression in response to radiation. Furthermore, the high expression of GRP78 in different types of cancers has been associated with RR. GRP78 overexpression increases DSB DNA repair and autophagy, as well as decreases apoptosis of tumor cells (13). Cetuximab is a monoclonal antibody used for the inhibition of EGFR and radiosensitization of tumor cells. However, it can also decrease the GRP78 expression of OPCC (13).

CSCs constitute a tumoral subpopulation with a high capacity for DNA repair, self-renewal, and differentiation towards other cell types and have been implicated in the recurrence of different types of tumors (114). CSCs present different mechanisms that have high resistance to different oncological therapies, including RT (115). In glioblastoma stem cells (GSC), an increase in ER luminal diameter, the activation of the UPR pathway, and the expression of proteins involved folding protein (such as GRP78 and GPR94) have been reported as mechanisms to avoid radiation-induced damage. Another survival mechanism in this tumor subpopulation is the activation of autophagy, which participates in the elimination of damaged cell fractions (116). The use of 2-deoxy-D-glucose (2-DG) may potentiate radiation-induced ERS to cytotoxic levels, inactivating the survival pathway and activating apoptosis (116).

Hypoxia is a feature frequently found in tumors, and its contribution to malignancy and treatment resistance has been demonstrated (117). Severe hypoxia also activates ER stress signaling. Particularly, the survival of a subset of hypoxic cells that determine tumor RR is dependent on the eIF2α-associated arm of the UPR. The eIF2α signaling promotes the synthesis of glutathione, cysteine uptake, and protection against ROS produced during periods of cycling hypoxia (118). In contrast, it has been reported that the enhancement of endoplasmic reticulum stress response under hypoxic conditions increases radiosensitivity in pancreatic and breast cancer cell lines *via* the stimulation of the insulin-like growth factor (IGF) signaling pathway and subsequent activation of the PI3K-mTOR pathway (119).

## Mitochondrial Adaptations to Radiation

**Figure 2:**
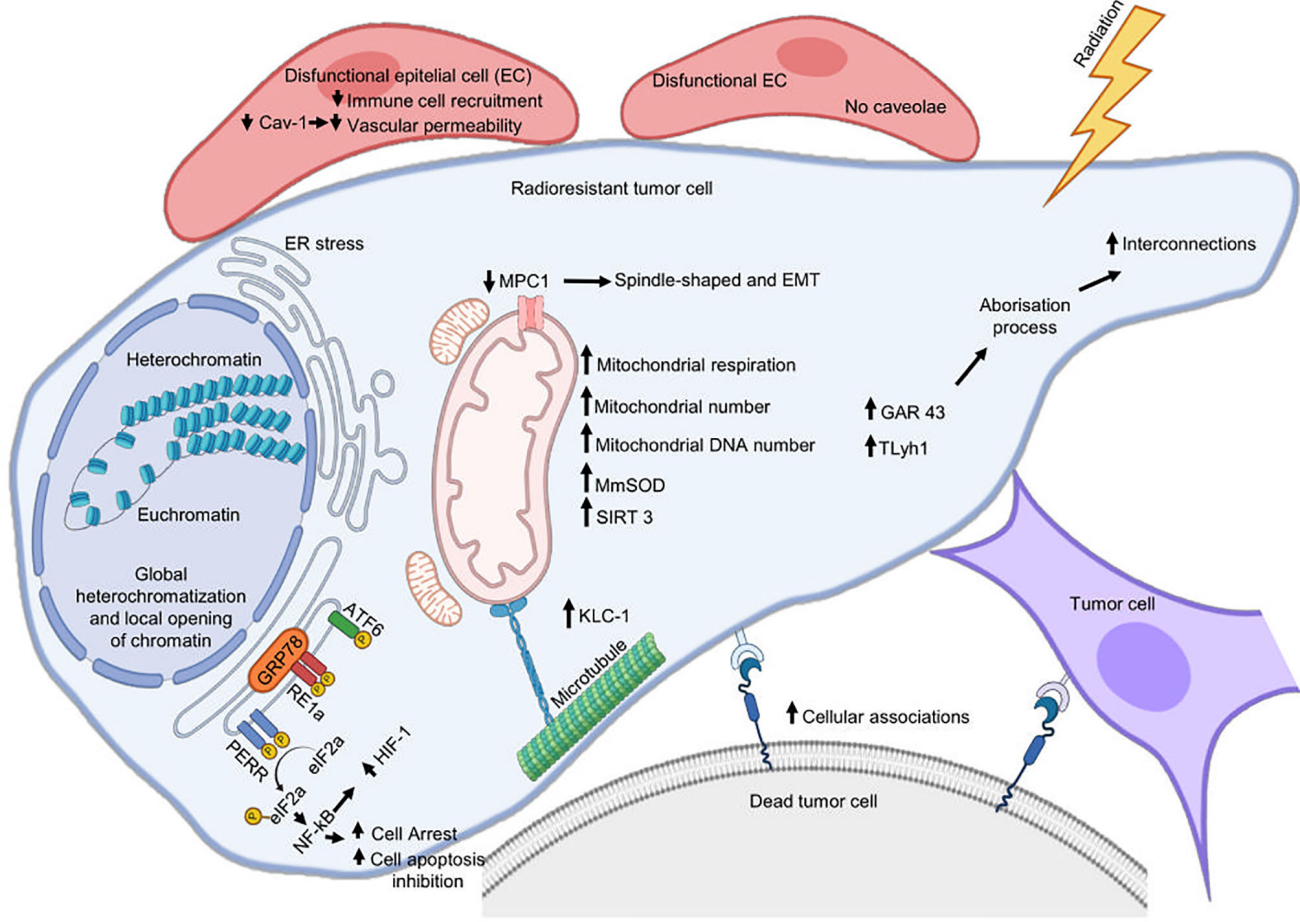
Cellular mechanisms associated with radioresistance. Cytoplasmic membrane, reticulum endoplasmic, and mitochondria are the main organelles where tumor cells assemble a response to develop radioresistance. Radiation can damage the endoplasmic reticulum (ER) homeostatic state and cause ER stress that will favor radioresistance. This last is also supported by mitochondrial alterations, metabolic remodeling, and by an increase in plasma membrane interconnections favoring the formation of cytoplasmic bridges. Cetuximab promotes radioresistance involving ERS pathway IRE1α/ATF6-GRP78. Silencing GRP78 inhibits the cooperative effects of radiotherapy and cetuximab inhibiting DSB repair and autophagy in OPCC. IRE1 promotes radioresistance in HPV-negative OPCC through IL-6 activation. Decreased MPC1 expression favors EMT and promotes radioresistance of cancer.

Mitochondria generate the chemical energy that cells need to carry out their biochemical functions through oxidative phosphorylation, the most efficient cellular pathway for the generation of ATP (120). The structure and function of mitochondria are compromised during different types of stress, including RT, so mitochondria respond through different adaptive mechanisms to support RR and maintain organellar and cellular homeostasis (**Figure 2**).

Lebeau et al. reported that acute stress in ER can also alter the mitochondria structure, promoting elongation and fragmentation. In response to ERS, mitochondria turn on a prosurvival mechanism called stress-induced mitochondrial hyperfusion (SIMH), avoiding premature fragmentation, stimulating metabolic activity, and facilitating adaptation and recovery during stress periods. In SIMH, ERS inhibits PERK-dependent eIF2α phosphorylation, decreasing translation, translocation, and accumulation of misfolded or damaged proteins in the mitochondrial lumen, thus maintaining cellular proteostasis (121).

Lynam et al. compared two esophageal adenocarcinoma cell lines with the same origin but with different degrees of RR, OE33 R, and OE33 P, to identify mitochondrial alterations associated with RR. They observed that the resistant subline OE33 R presented an increase in ROS levels and more DNA mitochondrial mutations than the parental line OE33 P, an increase in the number and mass of mitochondria, and more elongated and condensed mitochondria. Likewise, OE33 R presented bioenergetic alterations, such as increased mitochondrial respiration and oxidative phosphorylation and increased levels of intracellular ATP. Additionally, five genes involved in energy metabolism (ATP5G1, ATP5G3, ATPV0A2, NDUFC2, and NDUFS3) were overexpressed in OE33 R cells, supporting increased metabolic activity in these cells. Interestingly, radioresistant cells show an increase in their metabolic plasticity, changing from glycolysis to oxidative phosphorylation pathways, accompanied by enhanced survival (122). In head and neck cancer cells, preservation of mitochondrial functions after radiation has also been associated with a change from a glycolytic to more oxidative metabolism, increased mitochondria number, and a higher mtDNA content (123). Recently, Montenegro et al. also reported that radiation-induced changes that favor oxidative metabolism and an increase in ATP production in breast cancer cells were mediated by S-adenosylmethionine SAM. SAM is a donor of methyl groups in transmethylation reactions, so an increase in its cellular concentration stimulates the activity of different cellular methylases and promotes the hypermethylation of other cellular proteins. In this way, protein arginine methyltransferase 1 (PRMT1) methylates the BRCA1 protein after radiation and stimulates its nuclear translocation favoring DSBs repair *via* HR and inhibiting apoptosis. Thus, protein methylation also plays an important role in defense of tumor cells against IR (124).

However, exposure of tumor cells to a brief low-oxygen environment (7% O2 for 3 h) decreases mitochondrial respiration, resulting in exacerbated glycolysis, high lactate concentrations, and an increase in RR. During acute hypoxic stress, tumor cells adapt their metabolism through HIF-1α, which modulates glycolytic genes, making them less dependent on oxygen and increasing survival (125). The survival of HIF-1α knockdown tumor cells under hypoxia conditions is lower and increases their response to RT because they maintain a more oxidative metabolism that requires oxygen consumption, and since there is not enough oxygen, they are more likely to die. Importantly, HIF-1α inhibition altered tumor metabolism in mice exposed to a low oxygen environment (7% O2 for 3 h), enhancing RT response but having minimal effect on tumors in air-breathing animals (10). Epperly et al. reported that after irradiation of tumor cells, the expression of HIF-1α, c-Myc, and Glucose transporter 1 (GLUT1) increased in a dose-dependent manner, promoting the transport of glucose into the cell and stimulating glycolysis (126).

The signal transducer and activator of transcription 1 (STAT1), in addition to its role as a transmitter of interferon (INF) signaling and pro-apoptotic tumor suppressor, has been associated with energy metabolism regulation. The STAT1 overexpression pathway confers RR and INF resistance. In contrast, STAT1 knockdown in tumors alters the expression of genes and proteins of oxidative phosphorylation, the citrate cycle, and glycolysis/gluconeogenesis (127). In STAT1 knockdown tumor xenografts, radiation predominantly suppresses the glycolysis/gluconeogenesis pathway without significant change in STAT1 wildtype tumor xenografts. The IR-induced energy deprivation of proliferating STAT1-suppressed tumor cells constitutes a potential mechanism of tumor radiosensitization (128).

A determining point for the performance of oxidative phosphorylation in the cell is the transport of cytoplasmic pyruvate to the mitochondria. Mitochondrial pyruvate carrier (MPC) is the protein responsible for pyruvate transport to the mitochondria (129), and the subexpression of this carrier in pancreatic cancer and CRC cell lines induces changes associated with EMT and RR. MPC1-suppressed cells change their morphology from oval to spindle shape, the levels of E-cadherin transcript decreased, fibronectin increased, and migration and their ability to withstand radiation increased. When MPC1-suppressed cells were cultured in a glutamine-deficient medium, the changes in the EMT markers were suppressed; this suggests that EMT-like phenotype can be stimulated with alternative use of energy substrates, such as glutamine, when the entry of pyruvate into the mitochondria is reduced, thus compensating for ATP production (130).

Mitochondrial permeability transition pore (MPTP) is a non-specific pore located in the inner mitochondrial membrane, which opens under stress conditions resulting in alterations in oxidative phosphorylation, ATP depletion, and cell death (131). In a mouse model, Zhang et al. observed that after radiation to the whole body, liver cells from radiosensitive mouse strain (BALB/c) showed lower mitochondrial copy number, and MPTP opened sooner than radioresistant mouse strain (C57BL/6). Interestingly, they also showed that radiation response was maternally inherited (132).

The exact role that mitophagy plays in response to radiation is still debated. However, some authors have proposed that this mechanism may help cells eliminate mitochondria damaged by treatment (133). Zheng et al. reported that Parkin-mediated mitophagy plays a relevant role in cellular homeostasis maintenance and RR of breast cancer cells under hypoxic conditions. Under normal conditions, Parkin protein accumulation in dysfunctional mitochondria initiates the process of mitophagy. However, this process is inhibited by p53 protein. Parkin-p53 interaction inhibits the translocation of Parkin to the mitochondria, disrupting the protective mitophagy process and radiosensitizing cells significantly. However, in different types of tumors, there is a dysfunction of p53 (mutation or silencing), and so an increase in mitophagy (134). Additionally, mitophagy was markedly increased by low oxygen tension. Thus, these two facts could explain why p53-deficient cells adapt better to hypoxic stress conditions and are more radioresistant (134).

Kinesins are motor proteins associated with microtubules of the cytoskeleton involved in the intracellular transport of different cellular components, such as organelles and vesicles. Loss of Kinesin light chain 4 (KLC4) promotes apoptotic cell death and a decreased tumor growth in a mouse xenograft model. Also, downregulated-KLC4 cells have mitochondrial dysfunction through impaired mitochondrial respiration and an increase in ROS and mitochondrial calcium uptake. Because KLC4 is overexpressed in radioresistant lung cancer cell lines and tissues from lung cancer patients, it could be favoring mitochondrial homeostasis and the survival of tumor cells (135).

Mitochondria is the major source of ROS, which can cause oxidative damage to a wide range of molecules affecting cellular homeostasis; additionally, as we already mentioned, RT can also promote ROS generation (74). Manganese superoxide dismutase (MnSOD) is the major ROS-detoxifying enzyme located in the mitochondria; alterations in this enzyme generate mitochondrial and cellular dysfunction (136). Miar et al. have reported a higher expression of MnSOD compared to non-tumor samples in multiple tumor types, such as colon and lung, and an increase in MnSOD in middle-stage tumors of PCa. In addition, they also found high levels of MnSOD in all the metastatic tumors they analyzed, so overexpression of this enzyme may be involved in stimulating cancer hallmarks, such as migration and invasion, promoting thus carcinogenesis (137). Interestingly, it has been reported that MnSOD activity increases significantly after irradiation, contributing to the ROS neutralization and maintaining the cellular redox balance. In addition, irradiated cancer cells that overexpress MnSOD show an increase in the activation of the G2 phase of the cell cycle, so they can survive and divide despite the stress generated by radiation (126, 138).

On the other hand, it has been shown that higher doses of radiation generate lower mitochondrial membrane potential. Since the mitochondria use this membrane potential to generate energy in ATP form, its prolonged decrease can generate adverse effects on cells and lead to cell death (139). Epperly et al. reported that MnSOD overexpression in cancer cells stabilizes the initial changes in membrane potential generated by radiation, where another antioxidant enzyme, mitochondrial catalase, could maintain homeostasis at later times (83, 126).

Another mechanism of RR mediated by IL6 was studied by Tamari et al. comparing rat glioma cell lines (C6) as tumor cells against a rat astrocyte cell line (RNB) as a non-tumor cell. After irradiation, the addition of IL-6 reduces ROS levels and superoxide concentration in mitochondria, thus increasing C6 cell survival (140).

Additionally, there are other mitochondrial and epigenetic mechanisms associated with tumor RR. SIRT3 is a mitochondrial NAD (+)-dependent deacetylase that promotes deacetylation of other mitochondrial proteins to maintain metabolic homeostasis and prevent cell aging. Liu et al. reported that the SIRT3 promoter is overexpressed in radiation-treated tumor cells, and the NF-κB transcription factor mediates their transactivation. After radiation, SIRT3 and Cyclin B1/CDK1 are overexpressed and translocated to the mitochondrial matrix, where SIRT3 is phosphorylated and activated by Cyclin B1/CDK1, thus promoting the deacetylation of mitochondrial proteins, such as MnSOD, p53, and NADUFA9. In this way, SIRT3 maintains the mitochondrial homeostasis and increases survival and adaptive RR in tumor cells (141).

## Extracellular Adaptations of Tumor Cells to Radiation

The behavior, progression, and response to different therapies of tumor cells are influenced by the type of molecules, cells, and conditions present in their surrounding environment, that is, by the tumor microenvironment (TME) (142). TME is very heterogeneous and consists of multiple elements, such as a diversity of infiltrating cells of the host, stroma cells, the vascular system, extracellular matrix (ECM), secreted soluble factors, and different surrounding types of non-malignant cells. Dynamic interactions of these components can promote tumor progression, migration, invasion, metastasis, and survival of tumor cells (143). Generally, solid tumor cells (e.g., ovary, lung, cervical, and colon) can be subjected to an oxygen concentration gradient, where low concentrations (hypoxia) can stimulate the malignant characteristics of tumor cells and resistance to RT (144). On the other hand, it has been described that acidic pH, lack of nutrients, and low oxygen concentrations promote deficient blood perfusion and, consequently, hypoxia within the TME (145). Hypoxia promotes sustained angiogenesis and the activation of new neovascularization mechanisms, such as vasculogenic mimicry, the latter induced through EMT phenotype and changes in gene expression (144–146).

EMT is a complex mechanism that allows solid tumor cells to suppress their epithelial characteristics and acquire a mesenchymal phenotype. During EMT, cells show morphological changes and adhesion and migration capacity, facilitating their detachment from the primary tumor and the invasion of other body regions, thus favoring metastasis and tumor progression. Interestingly, an association between EMT and the generation of CSCs has been widely reported, promoting the formation of new tumors (147).

In CRC cell lines, the radiation triggers molecular changes consistent with EMT, such as low expression of the epithelial marker E-cadherin and high expression of mesenchymal markers, such as vimentin, fibronectin, and the Snail Family Transcriptional Repressor 2 (SNAI2), increasing invasion, migration, and survival of the radiated cells (105). Another report, using the ESCC KYSE-150 cell line and a xenograft tumor model, showed that the irradiation of KYSE-150 cells stimulated the EMT phenotype and the acquisition of stemness-like properties. In addition, those cells undergo morphological changes from cuboidal to spindle-like shape and show high expression of WNT1 inducible signaling pathway (WISP1), a signaling protein associated with the ECM, which plays a role in the development of the EMT phenotype and RR through regulation of genes associated with EMT (148).

The ECM is a dynamic three-dimensional network of proteins (collagen, proteoglycans, laminin, and fibronectin) and non-cellular components of tissue (water, minerals) that serve, among other things, as a cellular niche, as the organizer of TME components, and provides scaffolding for intercellular communication (149). When growing surrounded or embedded in the ECM, tumor cells are highly influenced by their matrix components. Inversely, tumor cells induce changes in their surrounding ECM to modulate its development, progression, and response to therapy (150). As mentioned already, tumor cells grown in a 3D environment have increased resistance to stressors, such as IR, compared to 2D cultures; this phenomenon is known as cell-adhesion mediated radioresistance (CAM-RR). It has been observed in several cell lines from different types of cancer that IR stimulates changes in the plasma membrane components. For example, after radiation, fibronectin and β1-integrin are overexpressed. Also, the β1-integrin is reorganized into clusters. Therefore, these two components can interact, stimulating cell-matrix interactions; consequently, RR and survival are increased (151–153). These interactions also influence chromatin structure, stimulate heterochromatinization with the aforementioned implications, and promote changes in gene expression and cellular response to environmental stimuli (92). Bai et al. compared the gene expression patterns of sarcoma cells grown in 2D and 3D by microarray analysis. These authors also observed that genes involved in tumor cell adhesion (N- and E-cadherin), gap junction (connexins Cx26, Cx43, Cx45), and ECM remodeling (COL1A1, LOX, FN1, SNED1, ITGB1, and LAMA4) were overexpressed in 3D cultures, and so are potentially involved in RR (154).

The lysyl oxidase-like 2 (LOXL2) is a protein that catalyzes the cross-linking of collagen and elastin components in the ECM and has been reported to contribute to the development and progression of several cancer types. A high expression of LOXL2 has been observed in DU145 and PC3 androgen-independent cell lines (from castration-resistant PCa), compared to LNCaP and 22Rv1 androgen-dependent cell lines. LOXL2 inhibition promotes radiosensitivity in prostate cells and xenograft tumors by EMT reversion and increased apoptosis by caspase-3 activation (155). PC-3, DU145, and LNCaP cancer prostate cell lines treated with radiation acquire characteristics of the EMT phenotype and stemness-like properties and show structural changes, such as loss of the glandular morphology, vacuolated cytoplasm, pleomorphic nuclei, and enlarged cell size. Furthermore, they increase the activation of p-Chk1 and p-Chk2 proteins and turn on the PI3K/Akt/mTOR signaling pathway; both processes can contribute to the repair of radiation-induced damage in tumor cells (156). Another study performed in poorly differentiated hepatocellular carcinoma (HCC) showed an association between PI3K/AKT/mTOR signaling pathway activation through the protein 3-phosphoinositide-dependent protein kinase 1 (PDPK1) and an increase in stemness characteristics, EMT, metastasis, DDR, and RR (157). Konge et al. demonstrated that TGF-β-induced mammary epithelium cells promote EMT and CSCs generation, which are more radioresistant compared to breast cancer non-stem cells. In addition, CSCs populations present very few polyploid cells, a G2/M arrest phase cell cycle, free radical scavengers, and activation of the death receptor pathways (FasL, TRAIL, and TNF-α), contributing to acquired RR during EMT (158).

On the other hand, although RT is a localized treatment, it promotes cytokine expression and systemic release. Cytokines are small proteins secreted by multiple cell types, which fundamentally modulate the immune and inflammatory response, and as already mentioned, they could mediate the survival of tumors to radiation. Ellsworth et al. conducted a prospective study to evaluate changes in cytokine expression patterns in NSCLC patients undergoing radiation therapy and found that different cytokines changed their expression during RT, including sCD40l, IP-10, MIP-1β, CX3CL1, VEGF, GM-CSF, IL-12, IFN-γ, IL-1a, and VEGF, which could participate in the promotion, growth, and progression of tumors by suppressing factors of the immune system, adding thus another layer to the complex response to the IR (159).

## Potential Molecular Targets to Enhance Radiosensitivity of Cancer Cells

There is no universal method to detect RR in patients. However, after RT, if a reduction in tumor volume is not observed in the expected response time or even increases, RR is suspected. RR can also be clinically deduced in cases of tumor recurrence, that is if tumor reappearance is detected after RT (15, 20). Depending on the type of tumor, stage of development, and location, other clinical manifestations associated with RR may be observed in patients. For example, in PCa, if symptoms of urinary obstruction continue after treatment, or if in a laboratory test the patient again shows elevated serum prostatic antigen levels, RR is also suspected (160).

The knowledge generated in recent decades on the mechanisms of tumor resistance to RT has made it possible to identify different molecules that can be used as molecular markers of RR or as therapeutic targets to increase radiosensitivity. Different research groups have focused on the search for markers of resistance to RT; some traits proposed as RR predictors include the presence of oxidative stress markers, such as some reactive oxygen species that are produced during therapy, tissue hypoxia which is evidenced by vascularity and central necrosis in some tumors, presence of cancer cells close to blood vessels, as well as the expression of specific interleukins, such as IL-8 (161). More specific molecular markers related to mechanisms of cellular adaptation and resistance to radiation have been proposed. TME and EMT signatures, TGF-β, poly ADP-ribose polymerase (PARP-1), or certain chaperone proteins have been found in radiologically resistant PCa. Analysis of these markers in patients can allow oncologists to assess the initial response to therapy and propose a more appropriate therapeutic strategy for each patient (160, 161).

As previously mentioned, RR is the main obstacle to the success of radiotherapies, so different research groups are constantly working in the search for strategies that allow reducing the resistance of tumor cells to radiation, and thus be able to increase the success of therapies and favorably impact on the quality of life and survival of cancer patients. Because one of the main mechanisms of RR in different types of tumors is the overexpression of molecules involved in DDR and DNA repair, these molecules are among the most explored therapeutic targets. However, molecules that participate in other RR mechanisms, such as epigenetic modulation, chromatin remodeling, maintenance of organelle homeostasis, lipid and carbohydrate metabolism, apoptosis, EMT, and signal transduction, among others, have been identified. Decreasing these molecules during RT can be of great help to increase the response of patients to therapy.

In recent years, multiple molecule types have been developed (mainly chemical inhibitors or interference RNAs) that specifically inhibit or decrease the action of proteins involved in tumor RR, and when tested in preclinical studies (in cell cultures or animal models), have given promising results for the radiosensitization of cells from different tumor types, such as brain, lung, pancreas, colorectal, breast, oral, cervical, prostate, and liver (**Table 2**). Inhibitors could be applied to patients in combination with radiation to increase the response to RT; even the combination of protein inhibitors can help increase radiosensitization and the success of the therapies. Because RR is a complex process, where different cellular pathways and mechanisms are orchestrated to increase the survival and reproduction of tumor cells, strategies must be focused on combating multiple aspects of tumor cell biology. The inhibition of key RR players, that is, participating in different pathways or mechanisms, would be especially useful to interfere with the process from different angles. However, other aspects must be worked on in parallel, for example, the mechanisms of action of the inhibitors, activation and inactivation mechanisms, effective doses to increase their effectiveness and reduce possible collateral damage.

**Table 2:** Potential molecular targets to enhance radiosensitivity of cancer cells.

Target	Process	Radiosensitization experiments	References
53BP1	Involved in DNA repair *via* the NHEJ and HR pathways.	53BP1 is knocked down using specific shRNAs in GBM cell lines.	(162)
AKT	Involved in cell survival, growth, cancer progression, and DNA damage repair.	Treatment of radioresistant lung cancer cells with Diosmetin, an AKT Pathway Inhibitor.	(163)
APE1	Involved in DNA repair *via* BER pathway.	Analysis of glioma and pancreas cells lacking APE1. Treatment of radioresistant pancreatic cancer cells with Lucanthone, an APE1 inhibitor. APE1 is knocked down using specific shRNAs in pancreatic cells.	(47)
Artemis	Involved in DNA repair *via* the NHEJ pathway	Mouse embryonic fibroblasts (MEFs) from DNA-PKcs mutant mice	(164)
β1 integrin	Signal transduction	GCS or patient-derived GBM cell cultures treated with AIIB2, a specific antibody against β1 integrin, and JNK inhibitor SP600125.	(165)
β-catenin	Wnt/β-catenin pathway	Treatment of radioresistant ESCC with iCRT14, an β-catenin inhibitor.	(74)
BRG1	Chromatin remodeling	BRG1 negative mutant overexpression in colon, breast, and lung cancer cells. Xenograft colon tumors that overexpress the BRG1 negative mutant.	(71)
Catalase	ROS detoxifying	32D cl 3, a hematopoietic progenitor cell line, was transfected with mt-catalase-plasmid, that overexpressing mitochondrial catalase. Intratracheal injection of mt-catalase plasmid-liposome complexes in C57BL/6NHsd female mice and subsequent thoracic irradiation.	(126)
CHOP (C/EBP homologous protein)	UPR pathway and Autophagy	CHOP is knocked by RNAi in breast cancer cells.	(166)
CUX1	DDR response	CUX1 is knocked by siRNAs in radioresistant breast cancer cells and MEFs (mouse embryonic fibroblasts).	(45)
CXCL1	Inflammation and DNA repair	CXCL1 is knocked by shRNAs in radioresistant GBM cell lines. Xenograft tumors of ESCC cells in combination with CAFs (XRCC1 producing cells) are implanted and after treated with an CXCL1 antibody.	(27, 58)
DNA-PKcs	Involved in DNA repair *via* the NHEJ pathway	MEFs analysis from DNA-PKcs mutant mice.	(164)
EGFR	Cell proliferation and survival	Radioresistant human lung carcinoma cells treated with erlotinib or cetuximab EGFR inhibitors.	(96)
EPOR (Erythropoietin Receptor)	Cell cycle arrest and grow	Erythropoietin-induced glioma and cervical cancer cells treated with tyrphostin B42, an inhibitor of JAK2 tyrosine kinase activity. JAK2 is an effector of EPOR. EPOR knockdown in GBM.	(167, 168)
FHIT	DNA methylation	Transfection of oral cancer cells using FHIT-overexpressing cDNA myc-tag plasmid. Generation of radioresistant mouse xenograft tumors that overexpress FHIT	(169)
GADD45α	BER	GADD45α overexpression in X-ray-resistant HeLa cell line, by transfection with GADD45α plasmid.	(48)
GAP-43	Neuronal differentiation	Glioblastoma cells grown under stem conditions (GBMSCs) with a genetic knockdown of GAP-43.	(102)
G0S2 (G0/G1 Switch 2)	Lipid metabolism	Targeting G0S2 by shRNAs in GSCs.	(4)
GRP78	ERS endoplasmic reticulum stress	Targeting GRP78 by siRNAs in OPSCC cell lines. GRP78 upregulation with 2-Deoxy-D-Glucose (2-DG) in GSC.	(13, 116)
GSK-3β	Involved in DNA repair *via* the NHEJ and HR pathways.	Inhibition of GSK-3β in pancreatic cancer cells using LiCl, AR-A014418, or SB216763 GSK inhibitors. Targeting GSK-3β by siRNAs in pancreatic cancer cell lines.	(170, 171)
HDAC	Histone deacetylase	Inhibition of HDAC in human prostate cancer cell lines using suberoylanilide hydroxamic acid (SAHA). Inhibition of HDAC in radioresistant esophageal carcinoma cells lines using trichostatin A and sodium butyrate.	(172, 173)
HDAC6	Histone deacetylase	Inhibition of HDAC6 in radioresistant GSC using HDAC6i.	(174)
JNK (c-Jun N-terminal kinase)	UPR pathway and apoptosis	Inhibition of JNK in radioresistant breast cancer cell lines using SP600125.	(166)
KDMs containing a Jumomji C (JmjC) domain	DNA methylation	Inhibition of KDM in radioresistant lung cancer cell lines using JIB-04.	(175)
KLC4	Mitochondrial homeostasis	Targeting KLC4 by siRNAs in lung cancer cell lines. Generation of mouse xenograft tumors with lung cancer cells lacking KLC4.	(135)
Ku70	Involved in DNA repair *via* the NHEJ pathway	Ku70 negative mutant overexpression in embryonic stem cells.	(176)
LIG4	Involved in DNA repair *via* the NHEJ pathway	Inhibition of LIG4 in colorectal cancer cells using SCR7 inhibitor. Targeting LIG4 by shRNAs in colorectal cancer cell lines.	(55)
LOXL2	EMT phenotype	LOXL2 knockdown by shRNA in castration-resistant prostate cancer cells.	(155)
MGMT	DNA-methyltransferase	Targeting MGMT by siRNAs in breast cancer cells lines.	(177)
MnSOD	ROS detoxifying	Targeting MnSOD by siRNAs in human pancreatic cancer cell lines. Intratracheal injection of MnSOD-PL plasmid-liposome complexes (that overexpress MnSOD) in C57BL/6NHsd female mice and subsequent thoracic irradiation.	(126, 138)
MSI1	Involved in DNA repair *via* the HR pathway	Silencing of MSI1 by shRNA in MSI1-high-expressing radioresistant GBM cell line. Generation of mouse xenograft tumors with GMB cancer cells lacking MSI1.	(67)
NFBD1	Involved in DNA repair *via* the HR pathway	Silencing of NFBD1 by shRNA in radioresistant NPC cell line.	(65)
OGG1	Involved in DNA repair *via* the BER pathway	Silencing of OGG1 by siRNAs in CRC cell lines. Inhibition of OGG1 in CRC cell lines using Chembridge 5245457 and 5552704 inhibitors.	(46)
P53	Transcription	Transfection of NSCLC cells using p53-overexpressing pCDNA3.1-p53 plasmid.	(178, 179)
PARP-1	Involved in DNA repair *via* the BER pathway	Inhibition of PARP-1 in HPV- negative in OPSCC using Olaparib.	(43)
PDK1	Signal transduction	PDK1 inhibition by siRNAs in hepatocellular carcinoma (HCC). PDK1 inhibition by BX795 in HCC.	(157)
PERK	Endoplasmic reticulum stress	Silencing of PERK by siRNAs in OPCC cell lines.	(110)
PI3k/mTOR	Signaling pathway	Dual PI3K/mTOR inhibition with BEZ235 in patient-derived OSCC cells or prostate cancer cell lines. Treatment of OSCC cell lines with RAD001 inhibitor decreases the phosphorylation and activation of mTOR and increases the RR.	(50, 84)
PNKP (Polynucleotide Kinase 3’-Phosphatase)	Involved in DNA repair *via* the NHEJ pathway	Inhibition of PNKP in prostate adenocarcinoma cancer cell lines using A12B4C3 PNKP inhibitor.	(180)
Pol β	Involved in DNA repair *via* the BER pathway	Human adenocarcinoma cells or MEFs cell lines that grow in conditions of confluence and expressing a dominant negative of Pol β.	(181–183)
Rad51	Involved in DNA repair *via* the HR pathway	Cells treated with chronic hypoxia had a reduced RR. Knocking down Rad51 with siRNA to levels like the levels seen under chronic hypoxia also radiosensitizes human lung cancer cells.	(184)
RPA1	Involved in DNA repair *via* the HR pathway	Targeting RPA1 by shRNAs in radioresistant hypopharyngeal cancer cell.	(64)
SHP1	Cell cycle regulation	Targeting SHP1 by siRNAs in radioresistant NSCLC cells.	(86)
SOCS	Signal transduction	Ectopic expression of SOCS1 in GBM cells. Blocking SOCS3 expression (by expressing a dominant-negative STAT3) in GBM cells.	(185)
TGF-β receptor	Signal transduction	Inhibition of TGF-β receptor in radioresistant gastric cancer cells using LY2109761.	(186)
Topo II α (DNA Topoisomerase II α)	Replication and transcription	Treatment of radioresistant laryngeal squamous cancer cells with 5-aza-2’-deoxycytidine, a DNA methyltransferase inhibitor.	(187)
WISP1	EMT	ESCC KYSE-150R cell line was treated with WISP1-specific neutralizing antibody.	(148)
WntT7	Signal transduction	Overexpression of Wnt7a in NSCLC by pcDNA6-Wnt7a transfection.	(188)

## Concluding Remarks

Cancer is a group of diseases that cause high rates of mortality and morbidity worldwide. For a long time, multiple treatments have been developed to combat different types of cancer. RT is applied in more than 50% of cancer patients due to its various advantages: non-invasive, painless, localized, and with high controllability. Despite its broad effectiveness, some patients show resistance to therapy and tumor recurrence, with negative implications on patients’ quality of life and survival.

After radiation, tumor cells can turn on a complex molecular and cellular response to maintain the integrity of their genome and organelles. This response conjugates different signaling pathways, which allow sensing the lesions and activate a DNA damage response. Genes modulated in response to radiation can alter multiple biological events, mainly, a redistribution of the cell cycle, DNA repair pathways activation, reconfiguration (global and local) of chromatin, increase in their metabolic plasticity, changes in the lipid and protein composition of the plasma membrane, the formation of intercellular networks, a cytoprotective response to stress generated in organelles such as ER and mitochondria, apoptosis evasion, EMT, and CSCs generation. Simultaneously, changes in the tumor microenvironment and ECM reorganization can occur, increasing the probability of survival, reproduction, and adaptation to radiation of tumor populations. These events can stimulate the appearance of tumors with more aggressive characteristics that interfere with patients’ response to treatments and promote tumor recurrence.

The clinical response of patients to radiation is very heterogeneous; it depends on the type of therapy applied, of the intrinsic heterogeneity between tumor types and subtypes, to the genetic variants present in patients that make them more or less susceptible to RT (189–192). The knowledge generated in recent decades has allowed us to propose different combined and personalized strategies to increase the success of RT. However, the translation of this information to clinical practice requires even more in-depth and comprehensive knowledge. Therefore, it is essential to continue with the molecular studies that allow us to identify the vulnerabilities of radioresistant cells.

## Author Contributions

ÁC-R, MM-L, SR-G, CL-C, and OH-C organized and wrote the manuscript. MM-L wrote Radiation Therapy in Clinical Practice section. OH, ÁC-R, and MM-L wrote Tumor Cells Activate Signaling Pathways Involved in DNA-Damage Response to Survive Ionizing Radiation section. ÁC-R and CL-C wrote Cell Cycle Adaptations in Response to Radiation. SR-G and OH-C wrote Chromatin Remodeling as a Mechanism of Radiation Adaptation, Changes in the Plasma Membrane That Favor Radioresistance, Endoplasmic Reticulum Adaptations to Radiation, and Mitochondrial Adaptations to Radiation sections. ÁC-R and CL-C wrote Extracellular Adaptations of Tumor Cells to Radiation section. OH-C and MM-L wrote Targeting to Enhance Radiosensitivity section. All authors revised the last version of manuscript. Figures and Tables were designed and produced by ÁC-R, MM-L, SR-G, and OH-C. All authors contributed to the article and approved the submitted version.

## Conflict of Interest

The authors declare that the research was conducted in the absence of any commercial or financial relationships that could be construed as a potential conflict of interest.

## Publisher’s Note

All claims expressed in this article are solely those of the authors and do not necessarily represent those of their affiliated organizations, or those of the publisher, the editors and the reviewers. Any product that may be evaluated in this article, or claim that may be made by its manufacturer, is not guaranteed or endorsed by the publisher.
